# Bio-fertilizer applications from poultry slaughterhouses in subtropical agriculture – Interactions between soil structure and nitrate dynamics

**DOI:** 10.1016/j.heliyon.2024.e38295

**Published:** 2024-09-21

**Authors:** Jucimare Romaniw, Thiago M. Inagaki, João Carlos de Moraes Sá, Fabricia Ramos

**Affiliations:** a State University of Ponta Grossa, Department of Soil Science and Agricultural Engineering, Av. Carlos Cavalcanti 4748, 84030-900, Ponta Grossa, PR, Brazil; b Norwegian Institute of Bioeconomy Research (NIBIO), Department of Biogeochemistry and Soil Quality, Høgskoleveien 7, 1430, Ås, Norway; c Rattan Lal Carbon Center, College of Food, Agricultural and Environmental Sciences, The Ohio State University, 2021 Coffey Rd, 43210, Columbus, OH, USA

**Keywords:** Nitrogen mineralization, Soil organic matter, Fertilization, Circular economy, Poultry

## Abstract

The No-till system and organic fertilization combined can be a potential strategy to avoid nutrient leaching, as the soil structure plays a crucial role in retaining them. In this study, we evaluated the influence of different rates of a bio-fertilizer made of industrial organic waste (IOW) from a poultry slaughterhouse on the percolation and stocks of nitrate in disturbed and undisturbed soil samples collected from a subtropical no-till field in southern Brazil. In an incubation experiment, we performed a percolation experiment using lysimeters and simulated rainfall for 180 days and evaluated the remaining soil nitrate stock after the incubation period. We set up a completely randomized experiment with three replicates using four IOW rates (equivalent to 0, 2, 4, and 8 Mg ha^−1^) and two sample types: disturbed and undisturbed soils. Using the bio-fertilizer increased nitrate mineralization from 0.77 to 1.55 kg ha^−1^ day^−1^. Overall, the IOW application increased the amount of percolated nitrate, significantly influenced by the simulated rainfall (p < 0.01). The amount of water flushed through the lysimeters was significantly higher for the disturbed soils (p < 0.05, LSD test), suggesting that the loosened structure promoted a higher water flux. No differences were observed between undisturbed and disturbed samples for nitrate percolation, implying that the amount of nitrate in the liquid soil phase may be a more critical factor in determining nitrate leaching than the water flux. The disturbed samples presented significantly higher nitrate percolation with increasing IOW rates, regardless of precipitation. Stocks in the 0–5 cm depth were 6.6 kg ha^−1^ higher for undisturbed samples (p < 0.05, LSD test). This result suggests preserving the soil structure can significantly increase the nitrate stocks upon IOW application.

## 1 Introduction

The increase in meat production worldwide driven by population growth is a major environmental problem due to, among several factors, the inefficiency in recycling waste generation [1]. Brazil is a significant pork and poultry producer, contributing nearly 14 billion kg of meat annually [2]. Therefore, alternatives for better use of waste generated in the poultry industry are essential for sustainable food production. Likewise, synthetic inorganic N fertilizers in agriculture are an indispensable concern worldwide due to several economic and ecological problems caused by their excessive use [3]. It is estimated that an additional 27 to 63 million tons of N will be necessary by 2050 only for maize production worldwide [4]. As N sources for inorganic fertilizer production are limited, alternative fertilization methods are increasingly important for promoting more sustainable agriculture.

In this sense, many research groups have explored several approaches to recycling waste from slaughterhouses into fertilizers [[5], [6], [7]]. It is known that bio-fertilizers made from animal waste can benefit crop yield [8]. These benefits are also applied to soils, including improvements in soil physical attributes [5], soil fertility [9], and the promotion of C-offset and crop energy efficiency [10]. However, evaluating these emerging technologies in organic fertilizer production is still incipient worldwide, especially in tropical and subtropical regions. Therefore, the development of studies assessing the use of feedstocks in organic fertilizer production is indispensable for the development of a circular economy and sustainable agriculture. In addition, how these bio-fertilizers influence the N dynamics in soils, such as N mineralization and nitrate leaching, is poorly understood. Environmental management using N from organic waste is necessary to comprehend the factors and processes that influence the conversion rate of organic N into forms available for plant uptake.

Also, changes in soil structure driven by soil management are recognized to significantly influence the nitrogen dynamics in soil, which include nitrification and denitrification processes [[11], [12], [13]] and nitrate leaching [14,15]. In tropical and subtropical agriculture, the use of practices that preserve soil structure, such as no-till and a diverse crop system (e.g., use of cover crops), is crucial for sustaining food production [[16], [17], [18]]. Therefore, understanding how differences in soil structure can affect the performance of organic fertilization, especially regarding N mineralization and leaching, is crucial for promoting an effective strategy in implementing these practices. Still, these interactions are highly unknown in organic fertilization, especially in tropical and subtropical agriculture. Therefore, this study aimed to understand the influence of bio-fertilizers produced from poultry slaughterhouse waste on nitrogen mineralization and nitrate percolation using a lysimeter experiment in disturbed and undisturbed soil samples. We hypothesized that using bio-fertilizer could be a good nitrogen source for crops and that the combination with undisturbed soil could help avoid nitrogen leaching and increase nitrate stocks in the soil.

## 2 Material and methods

### 2.1 Soil samples

The soil used for this experiment was collected from the 0–20 cm layer of a 15-year no-tillage crop field from Fazenda Escola Capão da Onça located in Ponta Grossa, PR (25° 05 ′ S and 50° 03 'W). This no-till field was cultivated with corn (*Zea mays*) and soybean (*Glycine max*) during the summer and ryegrass (*Lolium multiflorum*) and wheat (*Triticum aestivum*) during winter.

The mean precipitation of the site is 1500 mm. The mean maximum and minimum temperatures are 24 and 13 °C, respectively. A detailed illustration of the mean temperatures and precipitation is shown in Table 1 [19]:

**Table 1:** Data series over 44 years in Ponta Grossa: monthly distribution of rainfall (bars) related to average Maximum Temperature and Minimum Temperature. For the months from January to December.

	Jan	Feb	Mar	Apr	May	Jun	Jul	Aug	Sep	Oct	Nov	Dec
Precipitation (mm)	188	155	136	105	118	115	95	80	134	149	120	150
Max. temperature (°C)	28	27	26	24	22	20	20	22	23	24	26	27
Min. Temperature (°C)	17	17	16	14	11	9	9	10	12	14	15	16

The soil is classified as Cambisol [20] with a sandy loam texture (665, 88, and 247 g kg^−1^ of sand, silt, and clay, respectively). Soil fertility and physical attributes of the sampled soil are shown in Table 2.

**Table 2:** Chemical attributes of the soil in the experimental area (Cambisol. Ponta Grossa - PR).

Depth. (cm)	pH^1^	H + Al ^(2)^	Al^+3 (3)^	Ca^+2 (4)^	Mg ^+2 (4)^	K^+ (4)^	CEC ^(5)^	P ^(4)^	C ^(6)^	N ^(6)^	Bulk Density
		cmol_c_ dm^−3^						mg dm^−3^	g kg^−1^		Mg m^−3^
0–5	4.86	6.62	0.09	3.58	1.63	0.36	12.2	52	18.8	1.38	1.25
5–10	4.7	7.27	0.17	1.47	1.47	0.25	10.4	40.1	11.1	0.9	1.40
10–20	4.6	7.15	0.21	2.23	1.24	0.19	10.8	30.7	8.9	0.81	1.36
0–20	4.58	7.18	0.19	2.51	1.29	0.24	11.2	38.1	11.9	1.03	1.34

The numbers on the lines represent the average of ten points collected in the experimental area. 1: pH extracted by CaCl_2_ (1: 1); 2: Extracted with 0.5 mol l^−1^ calcium acetate. pH 7.0 and determined by titration; 3: Extracted with 1 mol l^−1^ potassium chloride and determined by titration with NaOH; 4: extracted by Melich^−1^ solution; 5: potential CEC; 6: Average of nine points analyzed by dry combustion.

### 2.2 Characteristics of organic waste

The compound used has industrial organic residues (IOW) from slaughterhouses originating from the slaughter process of poultry and swine, which includes birds killed during transport, excrement, blood, hair, feathers, cartilage, skin, legs, beak, biological sludge from the lagoons of waste treatment and boiler ash. The organic waste passed through a reactor at a temperature of 120 °C and 2 bar pressure to sterilize the material. After cooling, the material was composted for three months. After the composting phase, the material was dried, packed in vacuum plastic bags, and stored in a cold chamber. The IOW's chemical characteristics were as follows: pH = 6.7, moisture content = 3.6 %. Nutrient concentrations in mg g^−1^ were as follow: C = 321, N = 47.2, P = 10.5, K = 9.5, Ca = 4.1, Mg = 3.5.

### 2.3 Nitrogen mineralization incubation experiment

We have performed an incubation experiment using disturbed soil samples to evaluate the influence of different rates of industrial organic waste (IOW) from poultry slaughterhouses on N mineralization rates. The sampled soil was sieved in a 2 mm mesh and placed in polyvinyl chloride containers 10 cm in diameter and 20 cm in height (Supplementary Fig. 2) and kept under Temperature between 25 and 28 °C to study the mineralization of the NO_3_^−^. The organic material (IOW) was placed on the soil surface (without mixing or turning) with rates equivalent to 0, 2, 4, and 8 Mg ha^−1^. Each flask contained 500 g of soil. We have used a completely randomized design with three replicates per treatment.

The containers were periodically irrigated, maintaining the field capacity at 70 % during the evaluation period. Moisture loss was controlled by periodically weighing each flask and adding water when necessary (Supplementary Fig. 2). We performed 11 soil samplings for nitrate analysis over 115 days (0, 7, 14, 21, 28, 42, 56, 70, 84, 98, and 115 days of incubation).

### 2.4 Lysimeter experiment for nitrate percolation in disturbed and undisturbed samples

We performed an incubation experiment using lysimeters for 180 days to evaluate the effect of IOW application on nitrate percolation using disturbed and undisturbed samples. The undisturbed samples were collected in polyvinyl chloride (PVC) tubes with dimensions of 25 cm in height and 7.5 cm in diameter, with the aid of a hydraulic jack, which introduced the column PVC up to 20 cm deep. We then manually excavated the area around the tube to remove it without disturbing its interior. The disturbed samples were collected with cutting shovels, air-dried, sieved in a 2 mm sieve, and inserted inside the same PVC tubes used for the undisturbed samples.

The experimental design was completely randomized in a factorial arrangement (2 × 4) with three replicates. The factors evaluated were as follows: 1) sample type (disturbed and undisturbed), and 2) four rates of IOW application (equivalent to 0, 2, 4, and 8 Mg ha^−1^). The rates were chosen as a practical application range for agricultural purposes and to understand their influence on N mineralization, leaching, and retention in soils. The IOW was placed over the top of the soil column for both disturbed and undisturbed samples. A filter consisting of a synthetic thermal blanket was placed in the upper and lower part of the soil column to reduce the percolation of the water through the tube walls. The bottom part was sealed with a PVC cover containing a filter with a 3 mm diameter hole in the center of the lid and an attached pipe to collect the percolated water. The upper part was covered with perforated aluminum foil to minimize water evaporation but allow gas exchange.

The incubation period was in full 180 days. We added deionized water to the soil columns, simulating the local precipitation regime. The amount of water was calculated according to the rainfall level of each month (149, 120, 188, 155, 136, and 105 mm for October, November, December, January, February, and March, respectively). Each monthly volume of water was divided into 4 weeks of application. The first addition was made seven days after applying the IOW. The percolated solution was collected and filtered using 8 μm paper filters and frozen for analyzing the nitrate content.

### 2.5 Remaining nitrate content after the incubation period

After the incubation period, the remaining soil columns were divided into three parts corresponding to 0–5, 5–10, and 10–20 cm depths. The nitrate content of each section was immediately measured after the incubation period.

### 2.6 Nitrate analysis

The determination of mineralized NO_3_^−^ in the soil was carried out by extraction through the 2 mol l^−1^ KCl solution, according to Tedesco et al. [21]. Briefly, 10 g of moist soil was mixed with 50 ml of the 2 mol l^−1^ KCl solution with manual shaking. After 24 h of resting, we filtered the solution with an 8 μm paper filter. In the filtered solution, nitrate was determined in a Continuous Injection Flow (FIA) device. The primary focus of this study was to assess the impact of organic fertilization on nitrate leaching, a critical environmental concern. Nitrate is the predominant form of nitrogen that readily leaches through the soil profile and contaminates groundwater. While total nitrogen provides a broader overview of nitrogen dynamics, isolating the specific effects of organic fertilization on nitrate leaching was essential for addressing our research objectives. We acknowledge the importance of total nitrogen analysis and suggest it can be incorporated into future studies to gain a more comprehensive understanding of nitrogen transformations.

### 2.7 Statistical analysis

The statistical model of the completely randomized design was applied in a factorial arrangement (2 × 4) to study the percolation of NO_3_^−^ with three replications. For cases of significant F, the LSD test was used to compare means between soil types (undisturbed and disturbed). For the rates of IOW, we performed regression analysis. The levels of significance tested were p < 0.05 and p < 0.01. The analysis was conducted with the software SISVAR [22].

## 3 Results

### 3.1 Nitrogen mineralization as a function of the bio-fertilizer application

**Fig. 1:**
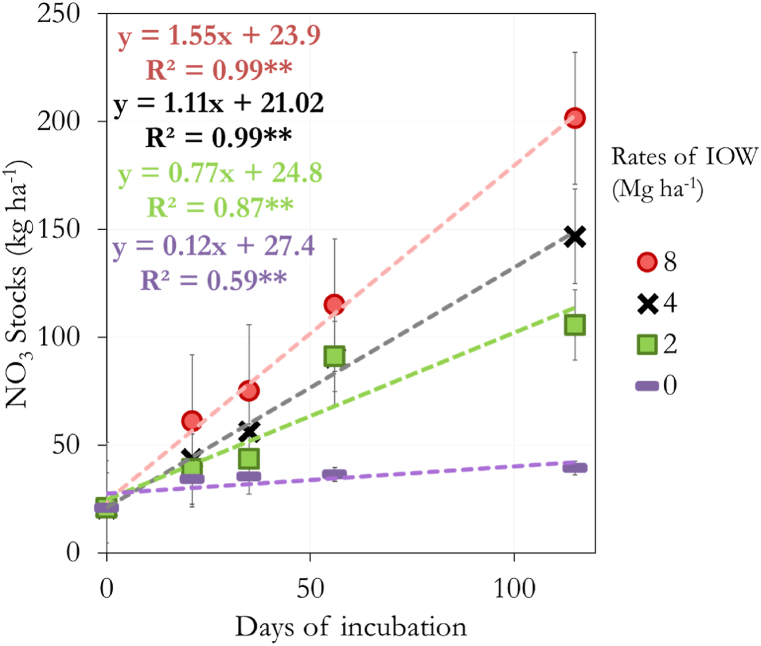
Nitrogen mineralization in disturbed soil with different Industrial Organic Waste (IOW) rates during a 115-day incubation experiment. ∗∗ = significant at p < 0.01. Each point is the average of three replicates. Error bars indicate standard error.

We have observed a linear increase (p < 0.01) of the NO_3_ stocks along the 120 days of incubation as a function of the bio-fertilizer applied (Fig. 1). Using the bio-fertilizer made from the industrial organic waste of poultry promoted an increase of nitrogen mineralization from 0.77 to 1.55 kg ha^−1^ day^−1,^ depending on the rate applied (Fig. 1). The control sample without IOW increased the NO_3_ by 0.12 kg ha^−1^ day^−1^.

### 3.2 Nitrate percolation in disturbed and undisturbed samples

**Fig. 2:**
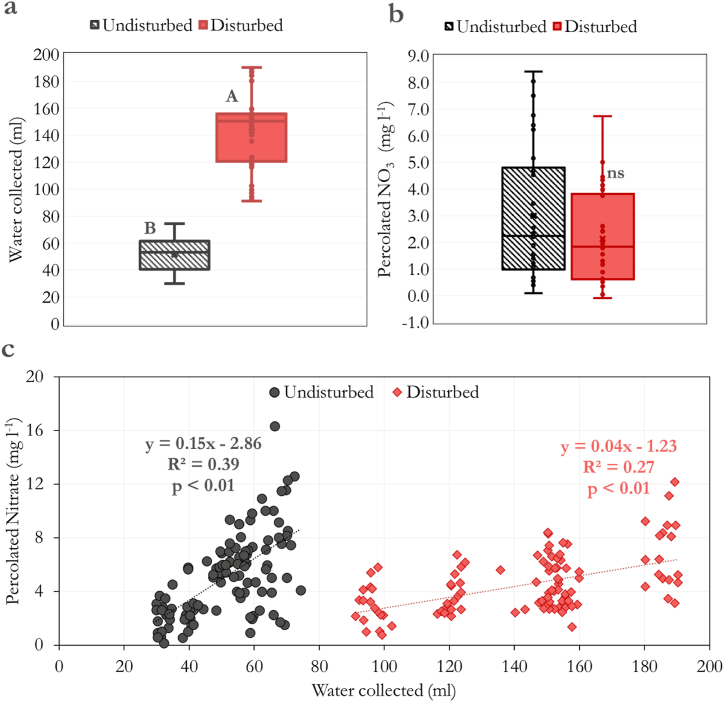
Comparison between undisturbed and disturbed samples in the average amount of water collected (a), percolated NO_3_ (b), and the relationship between both (c). The amount of water added weekly to simulate the local precipitation was as follows: 154, 124, 156, 195, 161, and 109 mm month^−1^ for the months from October to March, respectively. ∗∗: significant at p < 0.01, ns: non-significant. As the interaction between IOW rates and sample type was insignificant, all the IOW rates were accounted for when comparing disturbed and undisturbed samples.

The average amount of water that passed through the disturbed samples throughout the experiment was significantly higher than in the undisturbed ones (Fig. 2a). Nonetheless, the amount of NO_3_ percolated from both samples was the same, considering all the rates and months (Fig. 2b). We could see a significant relationship between these variables for disturbed and undisturbed samples (Fig. 2c). However, the slope of the undisturbed samples was higher than the disturbed, indicating a higher amount of percolated NO_3_ for a given volume of water (Fig. 2c).

**Fig. 3:**
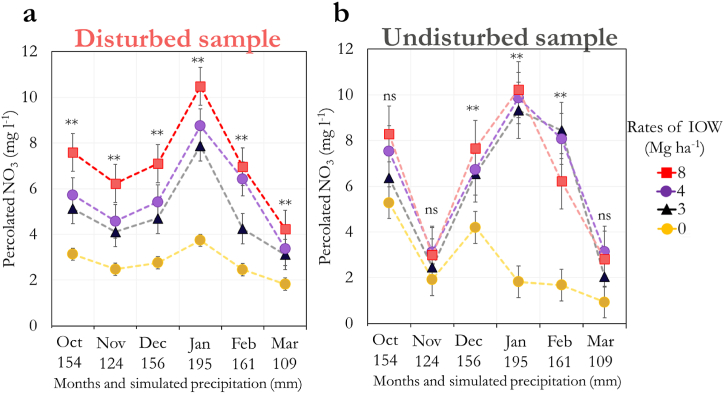
The amount of percolated nitrate under different rates of industrial slaughterhouse waste (IOW) along 180 days of percolation incubation (simulating the period from October to March) in disturbed (a) and undisturbed (b) samples. Both sample types were kept in PVC columns. The amount of water added weekly to simulate the local precipitation was as follows: 154, 124, 156, 195, 161, and 109 mm month^−1^ for the months from October to March, respectively. For a given month, each point is the average of three replicates. Error bars indicate standard error.

Despite the lack of a significant difference between disturbed and undisturbed samples for nitrate percolation, we have observed distinct dynamics between them during the experimental time, depending mainly on the simulated precipitation level (Fig. 3).

The rainiest months (December, January, and February) promoted a higher nitrate percolation in the experiment for both samples (Fig. 3). Nitrate percolation increased overall with the IOW rate applied, especially for disturbed soil samples where we observed increased levels of nitrate percolation in response to IOW rates, regardless of the precipitation level simulated (Fig. 3a). For the undisturbed samples, we have observed less difference between the IOW rates, but they have also promoted increases in nitrate percolation compared to the control (Fig. 3b)

**Fig. 4:**
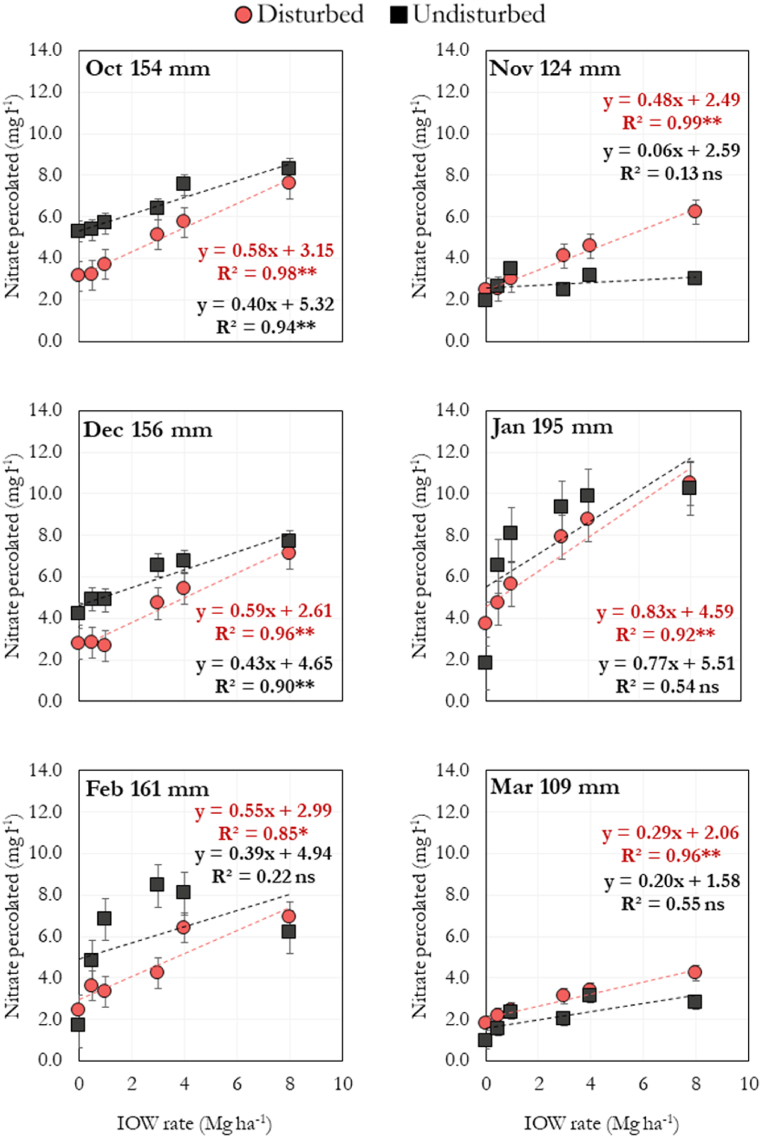
Correlation between industrial organic waste (IOW) rates and nitrate percolation along 180 days of incubation simulating the period from October to March in disturbed and undisturbed samples. Both sample types were kept in PVC columns. The amount of water added weekly to simulate the local precipitation was as follows: 154, 124, 156, 195, 161, and 109 mm month^−1^ for the months from October to March, respectively. ∗ significant at p < 0.05, ∗∗ significant at p < 0.01, ns = not significant. Each point is the average of three replicates. Error bars indicate standard error.

The increases in nitrate percolation in response to the IOW applications were linear overall for disturbed and undisturbed samples every month throughout the incubation period (Fig. 4). On average, every Mg ha^−1^ of IOW applied increased by 0.55 and 0.37 mg l^−1^ of nitrate percolation in disturbed and undisturbed samples, respectively.

### 3.3 Nitrate stocks upon industrial organic waste (IOW) application in disturbed and undisturbed samples

**Fig. 5:**
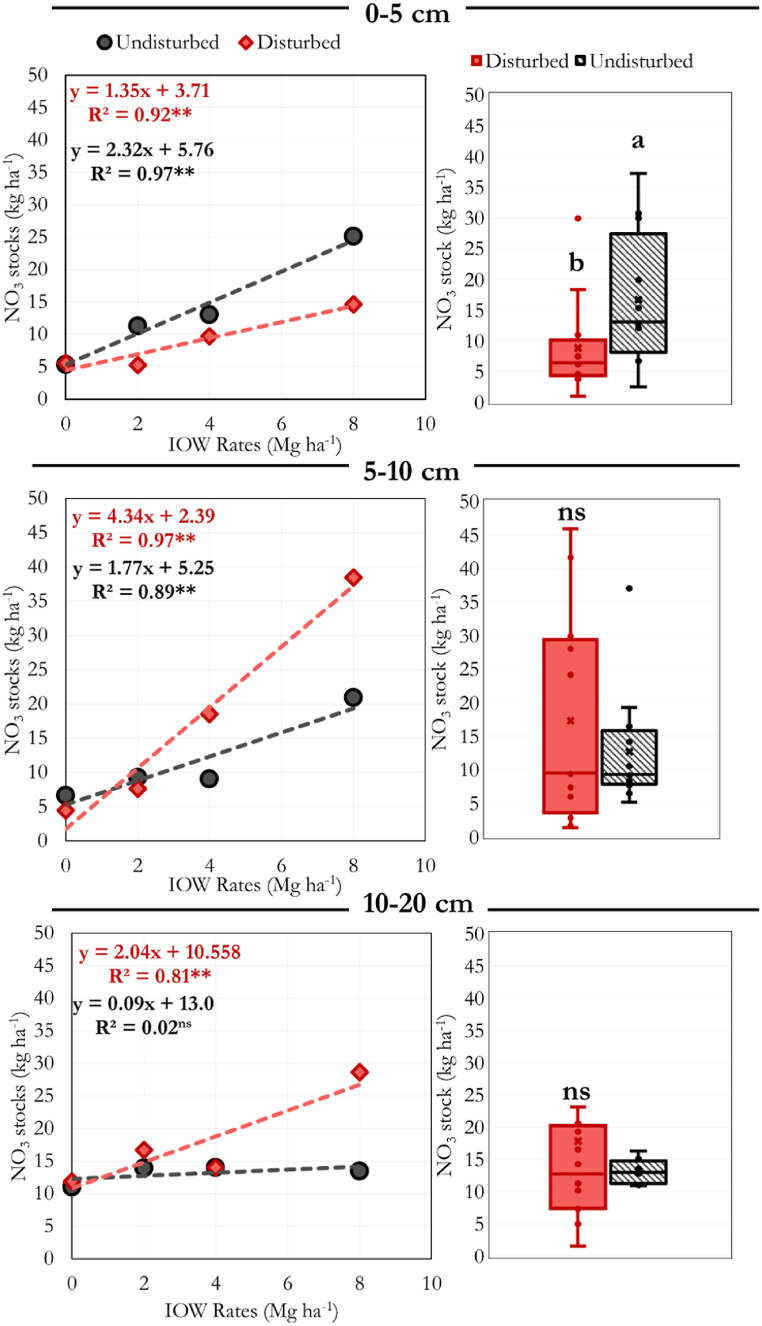
Soil nitrate stocks in disturbed and undisturbed samples in response to the addition of increasing rates of IOW (Control, 0.5, 1, 2, 4, and 8 Mg ha^−1^) after the incubation period (180 days). Soil columns were divided into the 0–5, 5–10, and 10–20 layers. ∗∗ = significant at p < 0.01, ns = not significant. As the interaction between IOW rates and sample type was insignificant, all the IOW rates were accounted for in comparing disturbed and undisturbed samples. Boxplots represent the data's third quartile, median, and first quartile range and comprise all the IOW rates. Each point is the average of three replicates. Error bars indicate standard error.

After the incubation period of 180 days, we observed linear increases in nitrate stocks in the soil in response to the rate of IOW applied, except for the undisturbed sample at 10–20 cm (Fig. 5). Despite the lack of differences between the disturbed and undisturbed samples in promoting nitrate leaching, we observed a significantly higher stock in the undisturbed samples at a depth of 0–5 cm (LSD test, p < 0.05). We have not observed significant differences between the disturbed and undisturbed samples for 5–10 and 10–20 cm depths.

## 4 Discussion

### 4.1 Dynamic of nitrate leaching on disturbed and undisturbed soils upon industrial organic waste (IOW) application

The N mineralization experiment demonstrated that the industrial organic waste (IOW) application provided significant amounts of nitrate to the soils with net release rates up to 1.43 kg ha^−1^ day^−1^ at the highest fertilization rates (8 Mg ha^−1^). Such results illustrate the capacity of organic fertilization as a source of crop nitrogen. As the mineralization study was done in a separate experiment from the leaching columns, the mineralization rates here are not directly used to interpret the amounts of leached nitrate. This study demonstrated that soil structure could significantly change soil responses to organic fertilization by directly comparing disturbed and undisturbed samples upon industrial organic waste (IOW) application. We have chosen to focus on nitrate concentrations rather than ammonium due to the lower relevance of ammonium leaching in subtropical conditions. This lower relevance happens mainly because nitrate is more mobile in soils than ammonium. Since nitrate is a negatively charged ion, it increases the repulsiveness in negatively charged soils compared with ammonium, which is positively charged [23]. For this reason, practices such as nitrification inhibitors are common in subtropical agriculture to avoid losses by nitrate leaching [24]. Also, due to the agronomic focus of our study, nitrate is a more relevant N form as it is the preferred form of nitrogen uptake by plants.

Undisturbed samples present a greater heterogeneity in structure compared to disturbed soils, which justifies the higher variations in the nitrate leaching results (Fig. 3b). Soil heterogeneity has been proposed to be a major influencer on the persistence of organic matter in soils by affecting the substrate accessibility to microorganisms (Lehmann et al., 2020). Our results illustrate this influence on the nitrate percolation dynamics upon organic fertilization. It is important to emphasize that despite the differences in their initial stage, the disturbed samples were also kept intact during the experimental time. Such a condition may have also contributed to avoiding further nitrate leaching, as it is commonly observed in tilled crop fields [25]. Furthermore, both sample types were collected from a 15-year no-till system field. Therefore, such simulations in our study mimic an eventual soil disturbance in a no-till area.

The loosened structure of the disturbed soils was likely the main factor contributing to the higher amount of water that passed through the columns in response to the simulated precipitation (Fig. 2a), as soil structure promotes a significant role in water retention (Otalvaro et al., 2016). Preserving soil physical properties is a major factor in elaborating strategies to avoid nitrogen losses by leaching. For example, it is well known that conservation agriculture practices that improve overall soil physics, such as cover crops and reduced tillage, can also contribute to reducing nitrogen leaching [[26], [27], [28]].

Because of the significant correlation between the water flow and the nitrate percolation (Fig. 2c) and the influence of precipitation on nitrate leaching [29], we expected the disturbed samples to present higher nitrate percolation than the undisturbed ones. Surprisingly, the nitrate percolation has mostly stayed the same between the different soil structures despite the high difference in water flow (Fig. 2b). The IOW rates applied were the same for both soils in our study. One possible reason could be nitrate adsorption to the soil particles, which may have preserved the nitrate in the disturbed soils despite the higher water flux rate. Nitrogen-rich organic matter is recognized to promote higher organo-mineral associations in soils [30,31]. Since Oxisols present a higher amount of mineral phase and Fe and Al oxides [32], such interactions may have significantly retained the fertilized-derived N in these soils. Since the disturbing may increase the surface area of soils, it may have enhanced this adsorption in disturbed soils, compensating for the higher water flow.

Therefore, the higher slope for the undisturbed samples in the correlation between water flow and nitrate percolation (Fig. 2c) suggests that the disturbed samples have not necessarily resulted in a higher nitrate leaching rate despite the higher flow. Thus, our results indicate that the amount of nitrate in the liquid phase of soil may be more critical in determining the leaching rates than the water flow intensity. For example, Investing in technologies that promote slow nitrogen release from fertilizers can be a promising strategy to avoid nitrate leaching in soils.

### 4.2 Increases of nitrate contents upon application of slaughterhouse industrial organic waste (IOW)

The significant increases in nitrate stocks upon applying IOW demonstrate the biofertilizer's efficiency in increasing this nutrient in the soil (Fig. 4). Despite the lack of differences in nitrate leaching, the significantly higher stocks in the undisturbed soils suggest that the intact structure may contribute to a higher retention of this nutrient, at least in surface depths.

Using the same IOW of this study, Romaniw et al. [10] demonstrated that this bio-fertilizer increased crop yields as much as conventional mineral fertilization, highlighting the use of the IOW to partially or fully replace the traditional NPK fertilizer. In this same study, the authors showed that the bio-fertilizer had a superior performance in grain energy balance compared to NPK and mixtures of both sources. Our results suggest that the high performance of the bio-fertilizer in increasing crop yields and grain energy balance, as demonstrated by Romaniw et al. [10], can be due to an efficient nitrate supply.

The higher nitrate stock observed in the 0–5 cm depth in the undisturbed sample relative to the disturbed suggests that preserving the soil structure can increase nitrate retention upon applying the bio-fertilizer. It is well known that the lack of soil disturbance in crop fields through conservation techniques such as the no-till system preserves the structure of soil aggregates [33]. As the undisturbed soils were sampled from a long-term no-till area (15 years) using soil columns, we assume that the soil structure conditions were overall preserved in these samples. Since the lack of soil disturbance is recognized to protect N from mineralization, especially in surface soil depths [34], it may have influenced the overall higher nitrate stocks in the 0–5 cm depth for undisturbed samples. The lack of difference between the disturbed and undisturbed samples for the depths of 5–10 and 10–20 cm suggests that the influence of soil disturbance on nitrate stocks upon bio-fertilizer application is mainly concentrated in the first centimeters of soil in this 180 days incubation experiment. Here, we focused on two significant processes of the nitrogen cycle: the leaching of nitrate and its retention in soil. These are two relevant processes for agriculture for agronomic reasons (e.g., N availability to plants in the form of nitrate) and environmental issues (e.g., potential contaminations of water bodies due to nitrate leaching). Future studies evaluating the influence of organic fertilization and soil structure could consider other processes of the N cycle, such as the influence of this interaction on nitrogen oxide emissions.

## 5 Conclusions

The biofertilizer application increased up to 10 times the N mineralization rate in the soil (0.12–1.55 kg ha^−1^ day^−1^). Nitrate percolation in the 20 cm columns was shown to be a function of the water flow caused by the simulated precipitation in the experiment. Disturbed samples presented a significantly higher water flow than undisturbed ones. However, we have not observed differences in nitrate percolation between the sample types. Such results suggest that factors related to nitrate release to the soil liquid phase may be more important than the water flow in determining nitrate leaching rates. Despite the lack of differences in nitrate percolation, undisturbed samples presented significantly higher nitrate stocks after the incubation period than disturbed samples at 0–5 cm depth. Overall, our results emphasize the potential of these bio-fertilizers as an alternative source to increase nitrate stocks in soil and demonstrate the influence of the soil structure in this dynamic.

## Data and code availability statement

Data will be made available on request.

## CRediT authorship contribution statement

**Jucimare Romaniw:** Writing – review & editing, Writing – original draft, Project administration, Methodology, Investigation, Conceptualization. **Thiago M. Inagaki:** Writing – review & editing, Writing – original draft, Conceptualization. **João Carlos de Moraes Sá:** Writing – review & editing, Writing – original draft, Supervision, Project administration, Investigation, Conceptualization. **Fabricia Ramos:** Methodology, Investigation.

## Declaration of competing interest

The authors declare that they have no known competing financial interests or personal relationships that could have appeared to influence the work reported in this paper.

## References

[1] Bujak J.W. (2015). New insights into waste management - meat industry. Renew Energ.

[2] Statista (2021).

[3] Sheoran S., Kumar S., Kumar P., Meena R.S., Rakshit S. (2021). Nitrogen fixation in maize: breeding opportunities. Theor. Appl. Genet..

[4] Alexandratos N., Bruinsma J. (2012).

[5] Auler A.C., Romaniw J., Sa J.C.M., Pires L.F., Hartman D.C., Inagaki T.M., Rosa J.A. (2020). Improvement in soil structure and water retention after application of industrial organic waste as a crop fertilizer. J. Soils Sediments.

[6] Bhunia S., Bhowmik A., Mukherjee J. (2019). 2019 International Conference on Energy Management for Green Environment (UEMGREEN).

[7] Roy M., Karmakar S., Debsarcar A., Sen P.K., Mukherjee J. (2013). Application of rural slaughterhouse waste as an organic fertilizer for pot cultivation of solanaceous vegetables in India. Int. J. Recycl. Org. Waste Agric..

[8] Ragályi P., Kádár I. (2012). Effect of organic fertilizers made from slaughterhouse wastes on yield of crops. Arch. Agron Soil Sci..

[9] Thomas B.W., Luo Y., Li C., Hao X. (2017). Utilizing composted beef cattle manure and slaughterhouse waste as nitrogen and phosphorus fertilizers for calcareous soil. Compost Sci. Util..

[10] Romaniw J., Sa J.C.D., Lal R., Ferreira A.D., Inagaki T.M., Briedis C., Goncalves D.R.P., Canalli L.B., Padilha A., Bressan P.T. (2021). C-offset and crop energy efficiency increase due industrial poultry waste use in long-term no-till soil minimizing environmental pollution. Environ. Pollut..

[11] Khdyer I.I., Cho C.M. (1983). Nitrification and denitrification of nitrogen fertilizers in a soil column. Soil Sci. Soc. Am. J..

[12] Schjønning P., Thomsen I.K., Petersen S.O., Kristensen K., Christensen B.T. (2011). Relating soil microbial activity to water content and tillage-induced differences in soil structure. Geoderma.

[13] Seifert J. (1962). The influence of the soil structure and moisture content on the number of bacteria and the degree of nitrification. Folia Microbiol (Praha).

[14] Finke P.A. (1993). Field-scale variability of soil-structure and its impact on crop growth and nitrate leaching in the analysis of fertilizing scenarios. Geoderma.

[15] Munkholm L.J., Hansen E.M., Olesen J.E. (2008). The effect of tillage intensity on soil structure and winter wheat root/shoot growth. Soil Use Manag..

[16] de Moraes Sá J.C., Lal R., Briedis C., de Oliveira Ferreira A., Tivet F., Inagaki T.M., Goncalves D.R.P., Canalli L.B., Dos Santos J.B., Romaniw J. (2022). Can C-budget of natural capital be restored through conservation agriculture in a tropical and subtropical environment?. Environ. Pollut..

[17] de Oliveira Ferreira A., de Moraes Sa J.C., Lal R., Tivet F., Briedis C., Inagaki T.M., Goncalves D.R.P., Romaniw J. (2018). Macroaggregation and soil organic carbon restoration in a highly weathered Brazilian Oxisol after two decades under no-till. Sci. Total Environ..

[18] Inagaki T.M., de Moraes Sá J.C., Tormena C.A., Dranski A., Muchalak A., Briedis C., de Oliveira Ferreira A., Giarola N.F., da Silva A.P. (2021). Mechanical and biological chiseling impacts on soil organic C stocks, root growth, and crop yield in a long-term no-till system. Soil Res..

[19] Nitsche P.R.C., Paulo Henrique Ricce, Wilian Da Silva, Pinto Larissa Fernandes Dias (2019). IAPAR.

[20] IUSS Working Group, W. (2015).

[21] Tedesco M.J., Gianello C., Bissani C.A., Bohnen H., Volkweiss S.J. (1995).

[22] Ferreira D. (2008).

[23] Greenberg A., Winkler R., Smith B.L., Liebman J.F. (1982). The negatively-charged nitrogen in ammonium ion and derived concepts of acidity, basicity, proton affinity, and ion energetics. J. Chem. Educ..

[24] Meng Y., Wang J.J., Wei Z., Dodla S.K., Fultz L.M., Gaston L.A., Xiao R., Park J.-h., Scaglia G. (2021). Nitrification inhibitors reduce nitrogen losses and improve soil health in a subtropical pastureland. Geoderma.

[25] Hansen E.M., Djurhuus J. (1997). Nitrate leaching as influenced by soil tillage and catch crop. Soil Res..

[26] Cameron K., Haynes R. (1986). Mineral Nitrogen in the Plant-Soil System.

[27] Matlou M., Haynes R. (2006). Soluble organic matter and microbial biomass C and N in soils under pasture and arable management and the leaching of organic C, N and nitrate in a lysimeter study. Appl. Soil Ecol..

[28] Nouri A., Lukas S., Singh S., Singh S., Machado S. (2022). When do cover crops reduce nitrate leaching? A global meta-analysis. Global Change Biol..

[29] Meisinger J.J., Ricigliano K.A. (2017). Nitrate leaching from winter cereal cover crops using undisturbed soil‐column lysimeters. J. Environ. Qual..

[30] Inagaki T.M., Possinger A.R., Grant K.E., Schweizer S.A., Mueller C.W., Derry L.A., Lehmann J., Kogel-Knabner I. (2020). Subsoil organo-mineral associations under contrasting climate conditions. Geochem. Cosmochim. Acta.

[31] Kopittke P.M., Hernandez-Soriano M.C., Dalal R.C., Finn D., Menzies N.W., Hoeschen C., Mueller C.W. (2018). Nitrogen-rich microbial products provide new organo-mineral associations for the stabilization of soil organic matter. Global Change Biol..

[32] Souza I.F., Archanjo B.S., Hurtarte L.C.C., Oliveros M.E., Gouvea C.P., Lidizio L.R., Achete C.A., Schaefer C.E.R., Silva I.R. (2017). Al-/Fe-(hydr)oxides-organic carbon associations in Oxisols - from ecosystems to submicron scales. Catena.

[33] Tivet F., Sa J.C.D., Lal R., Briedis C., Borszowskei P.R., dos Santos J.B., Farias A., Eurich G., Hartman D.D., Nadolny M., Bouzinac S., Seguy L. (2013). Aggregate C depletion by plowing and its restoration by diverse biomass-C inputs under no-till in sub-tropical and tropical regions of Brazil. Soil Tillage Res..

[34] Kristensen H.L., McCarty G.W., Meisinger J.J. (2000). Effects of soil structure disturbance on mineralization of organic soil nitrogen. Soil Sci. Soc. Am. J..

