# Supplementary material for: Bio-fertilizer applications from poultry slaughterhouses in subtropical agriculture – Interactions between soil structure and nitrate dynamics
Source: Heliyon. 2024 Sep 21;10(19):e38295. doi: 10.1016/j.heliyon.2024.e38295 (PMC11467538; doi:10.1016/j.heliyon.2024.e38295)
Supplement: Multimedia component 1 [file mmc1.docx]

Supplementary material for

**Bio-fertilizer Applications from Poultry Slaughterhouses in Subtropical Agriculture – Interactions Between Soil Structure and Nitrate Dynamics**

Jucimare Romaniw ^a^, Thiago M. Inagaki ^b *^, João Carlos de Moraes Sá ^c^, Fabricia Ramos ^a^

^a^ State University of Ponta Grossa, Department of Soil Science and Agricultural Engineering, Av. Carlos Cavalcanti 4748, 84030-900, Ponta Grossa, PR, Brazil.

^b^ Norwegian Institute of Bioeconomy Research (NIBIO), Department of Biogeochemistry and Soil Quality. Høgskoleveien 7, 1430 Ås, Norway.

^c^ Rattan Lal Carbon Center, College of Food, Agricultural and Environmental Sciences, The Ohio State University. 2021 Coffey Rd, 43210, Columbus-OH, USA


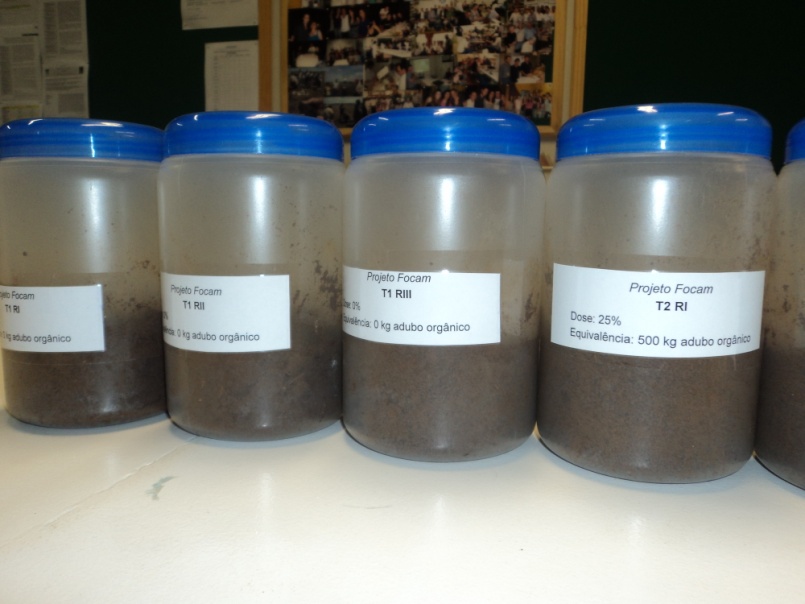


Supplementary Figure 1: Polypropylene flasks containing sieved soil with or without the addition of slaughterhouse residue used in the experiment to evaluate the mineralization of NO_3_-.


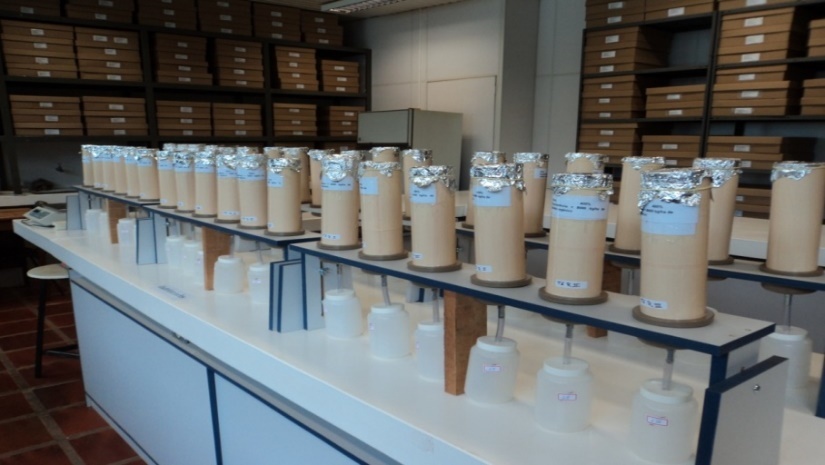


Supplementary Figure 2. Design of the experiment to assess nitrate percolation. PVC tubes containing samples of undisturbed or undisturbed soil with or without the addition of ROI in an NO_3_- percolation analysis experiment.
